# Enhancement and Evaluation of Interfacial Adhesion between Active Screen Plasma Surface-Functionalised Carbon Fibres and the Epoxy Substrate

**DOI:** 10.3390/polym14040824

**Published:** 2022-02-21

**Authors:** Yana Liang, Xiaoying Li, Mauro Giorcelli, Alberto Tagliaferro, Costas Charitidis, Hanshan Dong

**Affiliations:** 1 School of Metallurgy and Materials, University of Birmingham, Birmingham B15 2TT, UK; x.li.1@bham.ac.uk (X.L.); h.dong.20@bham.ac.uk (H.D.); 2 Department of Applied Science and Technology, Politecnico di Torino, C. so Duca degli Abruzzi 24, 10129 Turin, Italy; mauro.giorcelli@polito.it (M.G.); alberto.tagliaferro@polito.it (A.T.); 3 School of Chemical Engineering, National Technical University of Athens, 15780 Athens, Greece; charitidis@chemeng.ntua.gr

**Keywords:** carbon fibres, polymer-matrix composites, interface

## Abstract

This paper investigated the modification of the advanced active screen plasma (ASP) technology on PAN-derived carbon fibres (CFs) with gas mixtures of N_2_-H_2_ and N_2_-H_2_-Ar, separately. A more-than-30% improvement was found in the interfacial shear strength (IFSS) between the modified CFs and the epoxy substrate in the resulting composites, as disclosed by single fibre push-out tests. Based on the study of surface morphology, surface chemistry and water-sorption behaviour, the interfacial adhesion enhancement mechanisms were attributed to (1) the increased chemical bonding between the introduced functional groups on the fibre surface and the matrix; (2) the improved surface hydrophilicity of CFs; and (3) the enhanced van der Waals bonding due to the removal of surface contaminations.

## 1. Introduction

Owing to the excellent properties of carbon fibres (CFs), such as outstanding mechanical properties, high strength-to-weight ratio, high thermal stability and corrosion resistance, they have become the materials of choice for the reinforcements of high-performance composites [1]. However, their chemical inertness and low surface free energy, which is attributed to the high density of graphitic planes on the surface, limit the interfacial adhesion of CFs to the matrix [2]. This poses a critical challenge in further improving the properties of CF-reinforced composites to meet the requirements arising from demanding applications.

To this end, many surface modification methods have been developed to improve the interfacial adhesion of CFs to the matrix in composites, such as chemical treatment [3], electrochemical treatment [4], plasma treatment [5,6] and polymer/nanoparticles coating [7,8]. However, most of these treatments either lead to environmental concerns due to the use of toxic chemicals or reduce the mechanical properties of CFs due to surface damage.

Plasma treatment is attractive, owing to being less destructive, and having economic advantages and environmental benefits [9,10,11]. However, conventional plasmas can be harmful for the fibre strength mainly due to direct plasma damage [12]. Therefore, an advanced plasma technology, active screen plasma (ASP) that was developed based on the glow discharge plasma technology and the principle of the post plasma, has attracted great attention. In such a novel process, the entire workload is surrounded by a metal screen, on which a high-voltage cathodic potential is applied. Thus, the plasma is generated on the metal screen and not on the samples to be treated as in conventional plasma treatments, and the samples to be treated are at a floating potential. This setting allows more control over the physical and chemical interactions between the plasma and samples [13]. A preliminary study by Corujeira Gallo et al. [14] revealed a functionalised hydrophilic CF surface by ASP treatments, and researchers [15] recently discovered that the ASP treatment can lead to an increased single CF tensile strength. However, the interfacial shear strength between the ASP-treated CFs and the matrix has not been directly measured and the interfacial adhesion mechanism involved has not been fully investigated.

A series of methods have been developed to evaluate the effect of surface modification of CFs on the mechanical properties of the resulting composites and the CF/matrix interfacial adhesion. Notwithstanding the fact that improved mechanical properties of composites and CF/matrix interfacial adhesion by CF surface modification have been reported, the extent of the improvement spans a wide range. This could be attributed partially to the effect of different surface treatments on the strength of CFs and partially to the various evaluation methods with different stress states used.

The interfacial adhesion of CF-reinforced composites can be evaluated by direct examination of the interfacial shear strength (IFSS) of an individual fibre using the micro-bond test [16], single fibre pull-out test [16,17,18,19], single fibre push-out test [20,21,22] and single fibre fragmentation test [5,16,23,24]. However, except for the single fibre push-out test, all other tests are only applicable to model composites containing a single fibre [25]. Hence, it is difficult, if not impossible, to relate the measured IFSS from the model composites to the real performance of industrially manufactured composites. Hence, the single fibre push-out test is a desirable testing method and has been used in our previous work [19,26]. However, it remains challenging how to reduce the experimental errors and further improve the reliability of the results.

Therefore, the present work aimed to enhance the interfacial adhesion of PAN-derived CF-reinforced epoxy composites, evaluating the IFSS and advancing the scientific understanding of the mechanisms involved. To this end, advanced ASP technology was utilised to modify the surface of PAN-derived CFs using gas mixtures of nitrogen-hydrogen and nitrogen-hydrogen-argon. The nano-indentation-enabled single fibre push-out testing method was further improved by purposely designing the sample holder, using a conical indenter tip and statistically evaluating the data based on the Weibull distribution function. Systematic surface characterisation was carried out using scanning electron microscopy (SEM), X-ray photoelectron spectroscopy (XPS) and dynamic vapour sorption (DVS).

## 2. Material and Experimental

### 2.1. Materials

Poly-acrylonitrile (PAN)-derived epoxy-sized HTA40 E13 6K 400 tex CFs acquired from Toho Tenax^®^-E (Wuppertal, Germany) were used in this work. Their documented physical and mechanical properties are summarised in Table 1. A three-part epoxy-based resin system (Araldite^®^ LY 556, Aradur^®^ 917 and Accelerator^®^ DY 070) purchased from HUNTSMAN (Everberg, Belgium) was used as the matrix material for the samples prepared for push-out tests.

### 2.2. Active Screen Plasma Treatments

**Figure 1:**
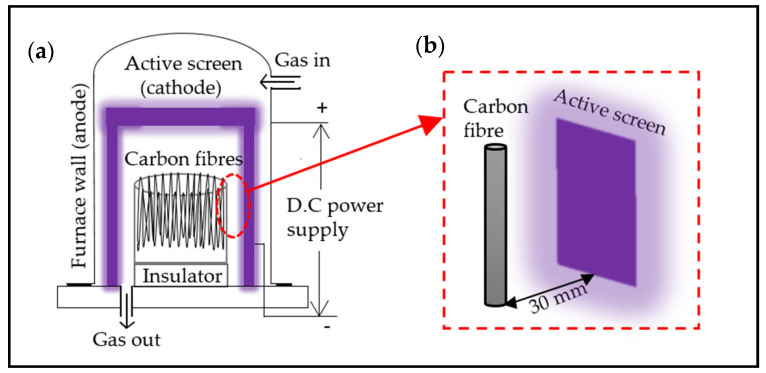
Schematic diagrams of (**a**) active screen plasma treatment and (**b**) carbon fibre arrangement [15].

The active screen plasma (ASP) treatments were conducted in an AS Plasma Metal 75 kVA + 15 KV industrial-scale unit equipped with an austenitic stainless-steel mesh cylinder cathode as the active screen (Figure 1a). The CFs were hung on a stainless-steel rack at a floating potential with a distance of 30 mm to the active screen (Figure 1b). The voltage controlled from 300 to 400 V was applied between the active screen (cathode) and the wall of the furnace (anode) in all processes. The treatment pressure was set as 75 Pa and the current was recorded to be between 60 and 70 A for all treatments. The temperature within the furnace was measured by a thermocouple placed next to the samples, and the temperature of all the treatments increased from room temperature (~25 °C) to about 32 °C. Two groups of ASP treatments with different gas mixtures were designed. The first treatment was conducted for 5 min in a gas mixture of 25% N_2_ and 75% H_2_ (ASPN), and the second treatment was carried out for 5 min with 10% Ar in the gas mixtures (ASPAr).

### 2.3. Scanning Electron Microscopy and X-ray Photoelectron Spectroscopy (XPS)

The surface morphologies of untreated and ASP-treated CFs were studied by scanning electron microscopy (SEM, JEOL 7000, JEOL Ltd., Tokyo, Japan). The surface composition was examined by X-ray photoelectron spectroscopy (XPS, PHI 5000 VersaProbe III, Roma, Italy) in a Thermo Scientific Sigma (Waltham, MA, USA) instrument with a monochromatic Al-X-ray source (1486.6 eV, 15 kV and 1 mA anode current). The resulting spectra were analysed using an in-house-developed Matlab^®^ software (2021a, Mathworks, Portola Valley, CA, USA) and the peaks were fitted using Gaussian-shaped components and a Shirley background.

### 2.4. Dynamic Vapour Sorption (DVS) Tests

A dynamic vapour sorption (DVS) gravimetric analyser (DVS advantage, Surface Measurement Systems Ltd, London, UK) was used to measure the adsorption characteristics of CFs. The absorbent mass was measured directly by a recording microbalance with a sensitivity of 0.1 μg housed in a controlled-temperature chamber. The pristine and the ASP CFs were cut into appropriate 5 mm lengths and then fed into the sample pan in the range from 10.0 to 10.5 mg. All the samples were first dried at 120 °C for 2 h and then went through the adsorption process carried out at room temperature (25 °C) for 10 h with a relative humidity of 60%, followed by the desorption process conducted at room temperature (25 °C) for 10 h with a relative humidity of 0%. The measurement was carried out immediately after and 2 months after the plasma treatments, in order to investigate the aging of the functionalised surfaces in air.

Further quantitative analysis of the DVS data was conducted based on the following calculations. The average water content (%) is defined based on the dry mass of the CFs as:

(1) $$Average\;water\;content=\frac{M_{ad/de}-M_{d}}{M_{d}},$$

where *M_ad_* is the average mass during the adsorption process, mg; *M_de_* is the average mass during the desorption process, mg; and *M_d_* is the dry weight after the preheat process, mg.

The chemisorption (%), which is the percentage of water molecules bonded directly with the functional groups, and the physisorption (%), i.e., the percentage of water molecules absorbed through the water–water interaction and evaporated after the desorption process, can be calculated as follows [28]:

(2) $$Chemisorption=\frac{M_{de}}{M_{d}},$$

(3) $$Physisorption=\frac{M_{ad}-M_{de}}{M_{d}},$$

### 2.5. Single Fibre Push-Out Tests

#### 2.5.1. Sample Preparation

Pristine and ASP-treated CFs were impregnated into the epoxy resin system (Araldite^®^ LY 556, Aradur^®^ 917 hardener and DY 070 accelerator in a ratio of 100:90:1). After being degassed in a vacuum desiccator, the CFs/epoxy resin systems were heated at 80 °C for 4 h (gelation process) and then heated at 140 °C for 8 h (post-cure) to form the composites for single fibre push-out testing.

Thin composite slices were cut from the produced composite bars with a thickness of about 1 mm. Then, the thin slices were glued onto the GATAN disc grinder for grinding and polishing with a sequence of silicon carbide papers of 1200, 2500 and 4000 grit and colloidal silica suspension (Struers OP-S) until the thickness of the composite slices was reduced to around 35 μm. The surface of the slices was observed by a microscope to guarantee that no damage was induced by the grinding and polishing procedures.

**Figure 2:**
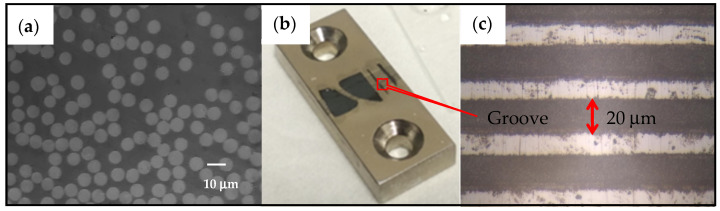
Images of (**a**) polished thin-composites plate, (**b**) thin composite plates mounted on the metal sample support and (**c**) magnified grooves made on the sample support.

As shown in Figure 2a, the fibres were observed to be surrounded by the resin without interface debonding. Then, the thinned composite slices were glued onto the top of a purposely designed metallic sample support, as shown in Figure 2b. Parallel narrow grooves with a width of 20 μm (Figure 2c) were cut into the surface of the metal support by a femtosecond laser beam. These narrow grooves can facilitate the push-out of single fibres from the composites while preventing the thin specimen slice from bending.

#### 2.5.2. Test Setup

**Figure 3:**
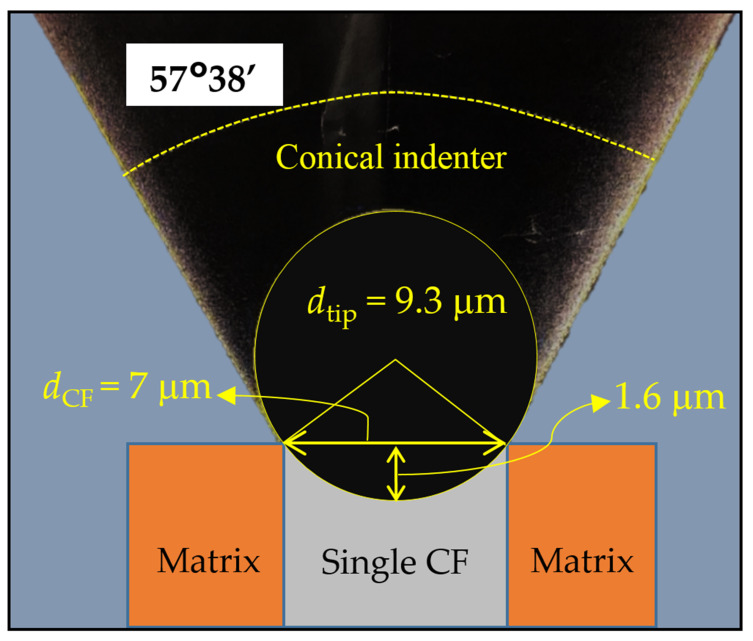
Geometry schematics of the conical indenter aligned with a single CF during push-out tests.

IFSS and the interaction between CFs and the surrounding resin were studied by micro push-out testing using a NanoTest Vantage system (Micro Materials Ltd., Wrexham, UK) with a diamond conical tip of 9.3 μm in diameter. The load was applied and released at a constant rate of 0.5 mN/s and the dwell period at the maximum load was set as 5 s for all tests. The individual fibre to be pushed out from the matrix was selected using a 400× optical microscope attached to the NanoTest system to represent the typical fibre-matrix distribution without any appreciable interface defects or damages. According to the geometries, as shown in Figure 3, the maximum indenter displacement before touching the adjacent matrix was calculated to be 1.6 μm when the indenter tip was precisely positioned to the centre of the fibre.

The thickness of the specimens was measured by using depth from focus by the high-resolution optical microscope during the push-out tests. The microscope was first focused on the surface of the metallic support and then on the top surface of the selected fibre by moving the lens away from the metal support. The displacement of the lens recorded by the system determined the local thickness, and it was measured for every test. After completing the push-out tests, the specimens and the most representative fibres were observed on both the top and back sides by SEM (JEOL 7000). The longitudinal cross-sectional cut off the pushed-out fibre with the resin aside by the dual-beam FIB-SEM (FEI Quanta 3D FEG) made it possible to view the interface between the carbon fibre and the matrix.

#### 2.5.3. Interpretation of a Typical Load–Displacement Curve

**Figure 4:**
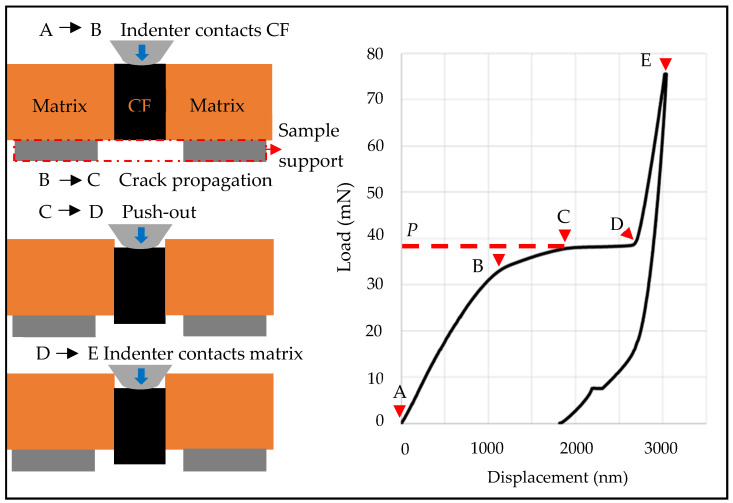
Schematic diagrams and typical load–displacement curve of push-out test.

The nano-indentation push-out test requires precise positioning and alignment of the indenter with the CF sample during the experiment. A typical load–displacement curve combined with the schematics of the test are shown in Figure 4, illustrating various stages of the push-out test. At the beginning of the initial linear part of the load–displacement curve (Point A), the conical indenter came into contact with the top surface of the CF. As the applied load increased (Points A to B), the indenter displacement increased to about 1.2 μm due to the elastic deformation of the fibre/matrix system and the plastic deformation of the fibre.

The following part of the curve (Points B to C) became less steep, which can be attributed to the decreased elastic modulus of the fibre/matrix system. Cracks could be initiated at the top of the specimen and were about to propagate along the CF/matrix interface. This stage is described as push-in by some researchers as a shallow step between the CF and the matrix could be observed during this stage [29]. The shear stress at the CF/matrix interface increased with the further increase in the applied load. When the load reached the critical value *P* at the point C, the elastic modulus of the CF/matrix system crashed, resulting in stress instability, and bringing a sudden increase in the displacement of the indenter tip, as evidenced by a plateau (Points C to D) in the load–displacement curve. The cracks between the CF and the matrix reached the bottom side of the specimen, resulting in a complete debonding between the fibre and the surrounding matrix. Then, the fibre was rapidly pushed out into the groove by the critical load *P*. The indenter tip solely contacted with the fibre and the displacement of the tip was about 2.7 μm at the end of this stage (Point D).

Further movement of the indenter tip led to a rapid load increase, as shown in the load–displacement curve (Points D to E), because the indenter tip started touching the adjacent matrix material. During this stage, the single fibre moved together with the adjacent matrix without the frictional sliding between them. After reaching the maximum load, the indenter started unloading and, finally, the load reduced back to zero. The final displacement of the indenter tip was around 1.8 μm, which exceeded the theoretical maximum displacement (1.6 μm) of this indenter tip. This can be attributed to the plastic deformation of the fibre/matrix system and the contact stiffness between the indenter and the specimen slice.

#### 2.5.4. Interfacial Shear Strength (IFSS)

##### Average Interfacial Shear Stress

The average interfacial shear stress (*τ*) at the fibre/matrix interface is given by the following equation,

*τ* = *P*/*πdh*, (4)

where *P* is the load at the instance when the interfacial sliding occurs (the plateau load in Figure 4) from the load–displacement curves, N; *d* is the diameter of the fibre, m; and *h* is the thickness of the sample, m. The fibre diameter was measured from the SEM micrographs for each tested sample.

##### Weibull Distribution

Although the failure mechanism of the fibre/matrix system is still a matter of debate, interface crack initiation and propagation are believed to be related to the randomly distributed defects on the CF surface and/or at the CF/matrix interface. Our experimental work has revealed a certain level of distribution for the measured critical load. Therefore, the weakest-link theory can be adopted and the Weibull distribution was applied in this research to statistically evaluate the probability of IFSS and to describe its scattering. The cumulative probability of failure is given by

(5) $$P=1-exp\left(-{\left(\tau_{s}/\tau_{0}\right)}^{m}\right),$$

where *τ_s_* is the shear strength, *τ*_0_ is the Weibull scaling parameter and *m* is the Weibull modulus.

## 3. Results and Discussion

### 3.1. Surface Morphology and Chemistry of CFs

**Figure 5:**
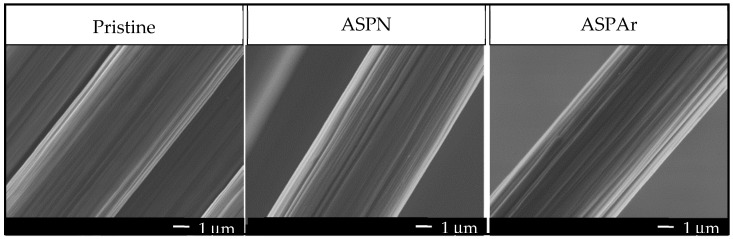
SEM micrographs of pristine, ASPN and ASPAr-treated CFs.

The surface morphologies of the pristine and the ASP-treated CFs are shown in Figure 5. Almost identical surfaces with longitudinal grooves can be observed in all cases due to the CF manufacturing process. Neither surface defects nor arcing damage was observed on the treated CF surfaces. This indicates that, due to the remote-plasma nature, ASP can effectively avoid surface damages caused by DC and other plasma technologies.

**Figure 6:**
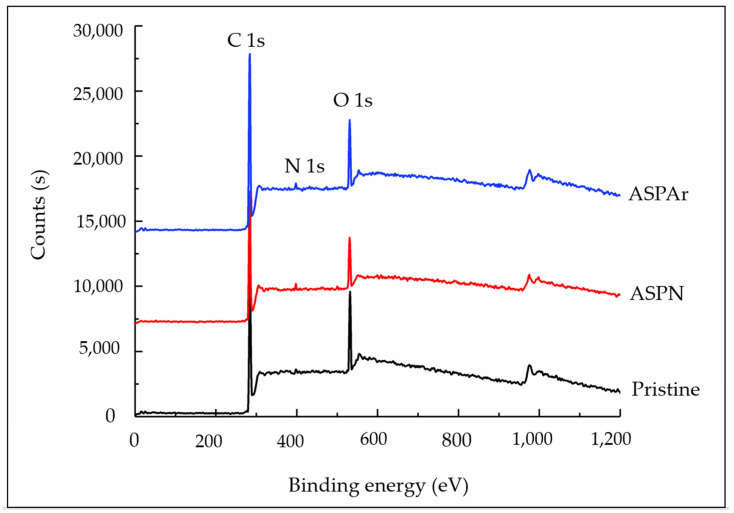
XPS surface survey spectra of pristine, ASPN and ASPAr-treated CFs.

The surface chemistry of the pristine and the ASP-treated CFs was examined by XPS, and the typical survey spectra in the binding energy ranging from 0 to 1200 eV are shown in Figure 6. The main resonance peaks are labelled C 1s, N 1s and O 1s. The peaks observed in the higher binding energy were attributed to the Auger effect [14]. The quantified analysis results in terms of the atomic content are summarised in Table 2, revealing a slight increase in the carbon content after both ASPN and ASPAr treatments. The nitrogen and the oxygen contents of the pristine CFs were attributed to the sizing layers, which can be largely removed by 5 min ASP treatments, as reported in our previous paper [15]. Thus, the increased nitrogen content of ASPN CFs was associated with the introduction of the N atoms by the ASPN treatments. By contrast, less nitrogen content was detected on the surface of the ASPAr CFs, because the nitrogen/hydrogen was partially replaced by argon during the ASPAr treatments. The oxygen contents on the surface of both the ASPN and the ASPAr-treated CFs are related to the remaining sizing on the surface of CFs and/or the oxidation of free radicals on the treated CF surfaces when exposed to air after the treatments.

**Figure 7:**
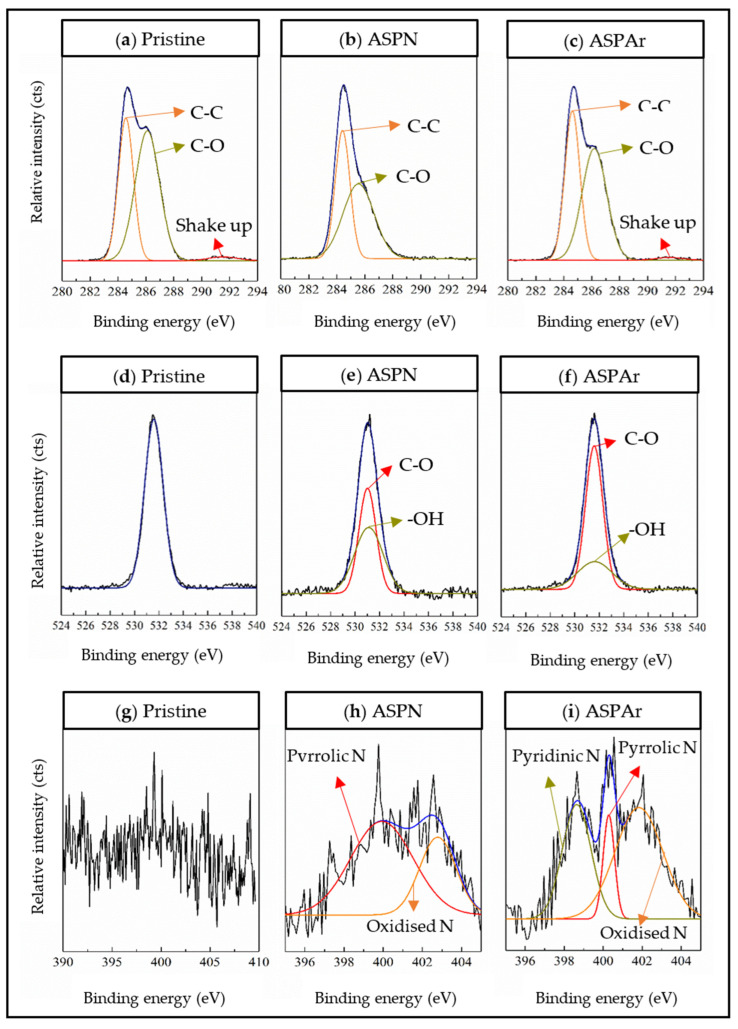
High-resolution XPS spectra deconvolution into surface functional groups for C 1s (**a**–**c**), O 1s (**d**–**f**) and N 1s (**g**–**i**) peaks area.

The high-resolution spectra corresponding to the C 1s, N 1s and O 1s regions of the pristine, ASPN and ASPAr CFs are depicted in Figure 7. The C 1s region could be deconvoluted into two functional groups C-C and C-O with the binding energies at about 284.5 and 286.2 eV, respectively. The O 1s region became wider after the ASP treatments, which can be assigned to the functional groups of C-O and -OH with the binding energies at about 531.1 and about 531.5 eV, respectively.

The most noticeable change in the XPS spectra was observed in the N 1s region, where the intensity of the signal increased after both the ASPN and the ASPAr treatments, and the shape of the peaks changed significantly. These changes could be attributed to the introduction of N-containing groups by the ASP treatments. As shown in Figure 7h,i, pyrrolic N and oxidised N groups were found on both ASPN and ASPAr-treated fibre surfaces with the binding energies at around 400.1 and 401.8–402.8 eV. This is because, during the ASP treatments, the CF surfaces were exposed to the energetic plasma species (ions, atoms, electrons, free radicals and other species), whose interactions contributed to creating active sites for the attachment of nitrogen species to form functional groups.

Pyridinic N with a binding energy of 398.6 eV was only found on the surfaces of ASPAr-treated CFs. The possible reason is the interaction of the nitrogen species with the active sites of vacant carbon bonds created by the collisions of argon species. It has also been reported that the formed pyrrolic N groups, located at the edges, voids and/or defects of the fibre structure, could be decomposed to form pyridinic N groups [30]. The introduced N-containing functional groups on the surface of CFs by ASP treatments, together with the O-containing functional groups, could react with the epoxy resin during the composites manufacturing, leading to the enhanced bonding of CFs to the epoxy resin.

### 3.2. Surface Water-Sorption Behaviour

**Figure 8:**
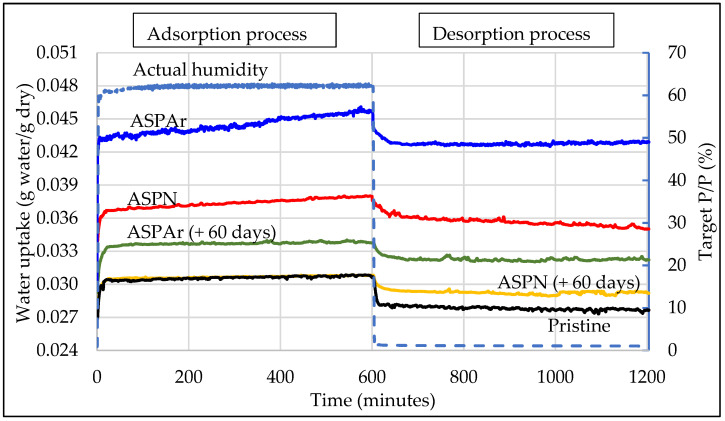
Dynamic vapour sorption and desorption of pristine and ASP-treated CFs within 20 h at room temperature (25 °C).

Figure 8 shows the measured water-uptake variations with time at relative humidities of 60% and 0% for the pristine and ASP-treated CFs, manifesting the adsorption process and desorption process. It can be seen that the actual relative humidity for the adsorption and desorption processes was very stable and slightly higher than the pre-set values of 60% and 0%.

The water sorption process was mainly dominated by the water-functional groups (chemisorption) and the water–water interactions (physisorption). That is, water clusters initially formed via hydrogen bonding on the functional groups, which acted as nucleating sites for water molecules to adsorb, followed by water cluster growth and filling micropores and coalescence. Thus, the adsorption parts of all CFs showed a steep increase with relative pressure at the beginning and then reached plateaus (adsorption isotherm), implying that all the chemisorption sites were occupied by water molecules and all the micro-pores and/or striations on the fibre surfaces were filled by water through capillary condensation. Then, as the relative humidity dropped to 0%, the water condensed and became unstable and desorbed by means of molecular evaporation, leaving only water molecules that were bonded directly to the chemisorption sites. Thus, the water-uptake decreased immediately at the beginning of the desorption process for all types of CFs and then reached another plateau, which were desorption isotherms.

Overall, all treated CFs showed a higher water-uptake compared to the pristine CFs. This is an indication that the ASP treatments increased the hydrophilicity of the CF surface, with the ASPAr treatments being more effective in enhancing the hydrophilicity of CFs than the ASPN treatments. Furthermore, even after exposure in air for 60 days, the ASP-treated CFs still exhibited improved hydrophilicity as compared with the pristine CFs. This improved hydrophilicity of ASP-treated CF surfaces could be attributed to the introduced functional groups and their surrounding carbon atoms, whose affinity towards water was increased because of the polarisation effects of introduced nitrogen (nitrogen and oxygen have a stronger electronegativity than carbon). The more hydrophilic surfaces resulted in a larger adsorptive capacity for water in the ASP-treated samples than that in the pristine CFs.

Further quantitative analysis was conducted based on Equations (1)–(3) and the results are summarised in Table 3. It can be seen that the average water content for the adsorption processes increased from 3.06% for pristine CFs to 3.74% for ASPN CFs and 4.46% for ASPAr CFs. After desorption processes, the average water content decreased to 2.79%, 3.57% and 4.28% for pristine, ASPN and ASPAr CFs, respectively. The chemisorption of water increased from 2.79% for pristine CFs to 3.57% for ASPN CFs and 4.28% for ASPAr CFs. Clearly, the ASP-treated CFs exhibited a greater water content and chemisorption of water molecules, which indicates that more water molecules were adsorbed on the ASP-treated CF surfaces than on the pristine CFs through hydrogen bonding with functional groups. This is supported by the XPS results that nitrogen-containing functional groups were introduced by ASP treatments. We can conclude that ASP treatments could introduce and/or increase the concentration of the functional groups on the CFs, which improved their affinity towards water molecules.

The ASP treatments with the addition of argon resulted in a higher chemisorption of water, though fewer nitrogen atoms were detected by XPS in these treatments. This is because the chemisorption capacity of water depends not only on the concentration of functional groups and their affinity towards water via hydrogen bonding, but also on the active sites that carry partial charge. Moreover, some experiments have also found that the relationship between various functional groups and water sorption is complicated. This could be attributed partially to the perfect match for hydrogen bond formation between the water molecules and the functional groups, and partially to the accessibility of the functional groups to water molecules [31]. Therefore, the increased chemisorption of the ASPAr CFs could be attributed to the addition of argon, which offered the potential of creating more active sites and forming a pyridinic N group.

The water-sorption behaviour of the ASP-treated CFs was also tested 60 days after the treatments to investigate the potential aging effect on the ASP-treated CFs. From the results shown in Figure 8, it can be seen that the aged ASP-treated CFs still exhibited better water-sorption properties than the pristine CFs but worse than the freshly ASP-treated CFs, indicating that the introduced functional groups underwent an aging process after being exposed to air for 60 days.

The physisorption decreased for both the freshly ASP-treated CFs and the aged ASP-treated CFs. This is believed to be associated with the sizing layers on the pristine CFs and the change in surface morphology caused by the mild chemical etching of hydrogen (Figure 5).

### 3.3. Interfacial Property of Carbon Fibre-Reinforced Composites

#### 3.3.1. Load–Displacement Curves and Post-Observation of Pushed-Out Fibres

**Figure 9:**
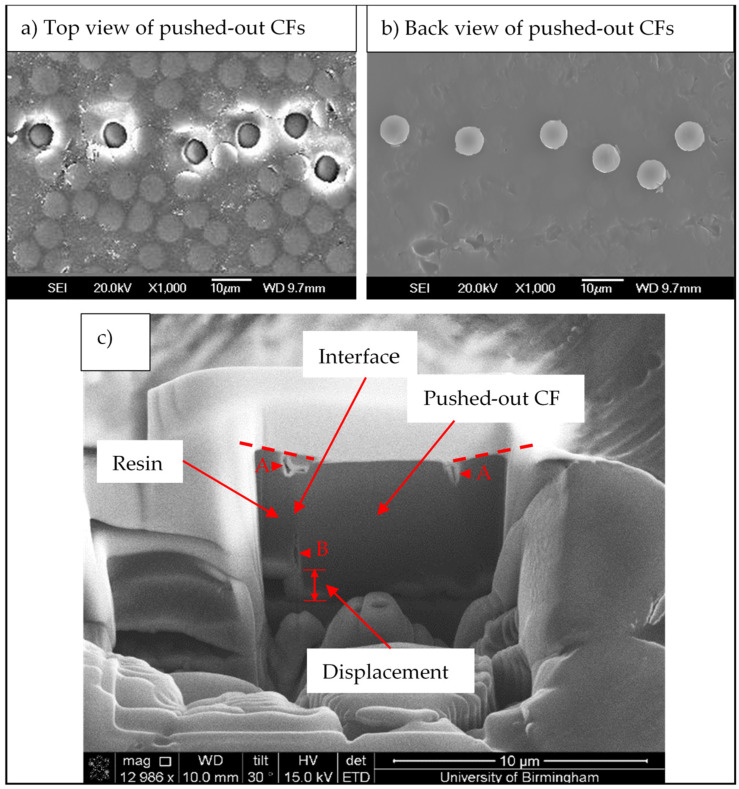
SEM observation of the push-out-tested micro-composite specimen: (**a**) top view and (**b**) back view of the pushed-out carbon fibre discs, and (**c**) FIB-produced cross-sectional view of a pushed-out CF disc.

The top and back sides of the tested composites were observed by SEM, and some typical micrographs are shown in Figure 9. It can be seen clearly from both the top (Figure 9a) and the back sides (Figure 9b) that the tested fibres were pushed out successfully. The pushed-out fibres were presented in a row because of the geometry of the grooves on the sample support. Furthermore, the longitudinal interface between the pushed-out CF and the matrix was examined by Focused Ion Beam (FIB)/SEM. As shown in Figure 9c, the fibre was pushed out, evidenced by a ~2 μm push-out step clearly shown between the matrix and the CF on the back side. In addition, plastic deformation on the adjacent matrix was revealed on the top side as the dashed line. Clear debonding and cracking were observed at the CF/matrix interfaces near the top surface of the CF (Sites A) and near the exit site of the matrix (Site B).

**Figure 10:**
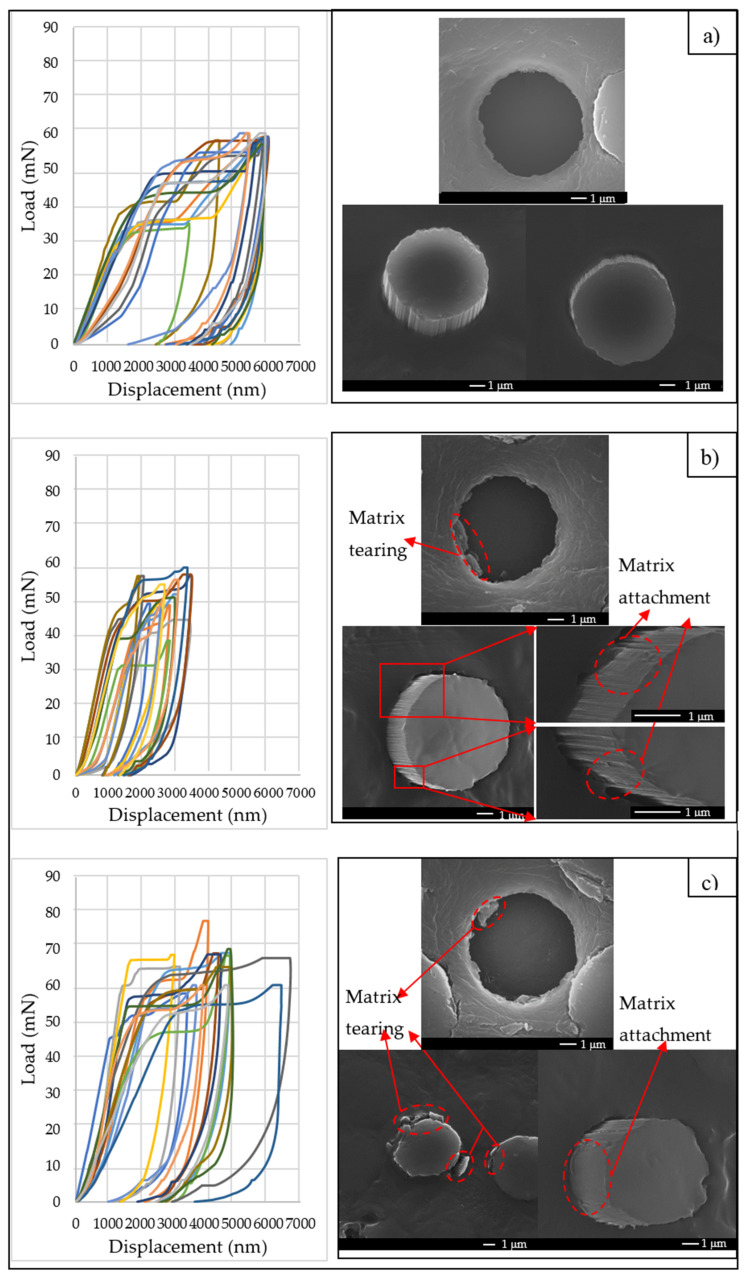
A group of force–displacement curves and SEM images of pushed-out CFs taken from composites made with (**a**) pristine CFs (*h* = 39.1 μm), (**b**) ASPN-treated CFs (*h* = 31.2 μm) and (**c**) ASPAr-treated CFs (*h* = 36.4 μm).

Figure 10 shows the force–displacement curves recorded during the micro-push-out tests of the thin composites made with pristine, ASPN and ASPAr CFs as the reinforcements, accompanied by detailed micrographs showing the typical views of the pushed-put fibres from the top and bottom side of the samples.

The critical load and the maximum displacement of each test can be identified from the recorded load–displacement curves. It should be pointed out that the thickness difference must be taken into consideration when comparing the maximum displacement, and the average sample thickness was 39.1 µm, 31.2 µm and 36.4 µm for the pristine, ASPN and ASPAr composites, respectively. From the morphologies of the pushed-out fibres, it can be seen that without plasma treatment, as shown in Figure 10a, the pushed-out pristine fibre showed a clear surface without clear evidence of appreciable fibre damage or transferred material from the matrix. The adjacent matrix surface was smooth and flat; neither macro sinking at the top surface nor bulging at the back surface by plastic yield could be observed. This implies that only elastic deformation may have occurred for the matrix during the push-out test.

**Figure 11:**
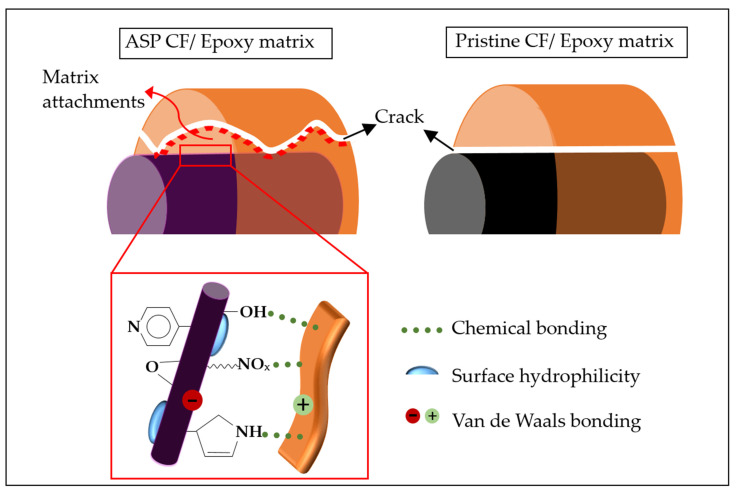
Schematic diagrams of interfacial failure mode of ASP CF/matrix and pristine CF/matrix.

By contrast, the response to push-out and the behaviour of the CF/matrix of the ASP CF-reinforced composites differed greatly from what was observed above for the pristine CF-reinforced composites. For the ASP-treated CF-reinforced composites, matrix material was transferred to and attached on the CF surface, as revealed by Figure 10b,c, indicating that in-matrix cracks were created. Adjacent matrix tearing was observed on the top side and, in some cases, the matrices were protruded together with the fibres on the back side, implying that local failure occurred within the matrices rather than at the CF/matrix interfaces. The corresponding interfacial failure modes of the ASP-modified CF/matrix and pristine CF/matrix are schematically illustrated in Figure 11. These observations indicate that the ASP-treated CFs had a strong interfacial adhesion with the epoxy resin in the composites. It can be also deduced that the CF/matrix interface shear strength would be higher than the shear strength of the matrix.

#### 3.3.2. Interfacial Shear Strength

The average IFSS was calculated according to Equation (4) and the results are summarised in Table 4, together with the diameters of the pristine and ASP-treated CFs. The results show very promising improvements of the IFSS of the composites made with ASP-modified CFs than the ones made with pristine CFs. The IFSS increased from 55 MPa to 72 MPa for ASPN and 77 MPa for ASPAr CF-reinforced composites, indicating an improvement exceeding 30%. However, there are relatively large variations in the measured average IFSS values, which makes it difficult to conclude that a significant improvement in the IFSS by ASP treatment of the CFs was obtained if the experimental errors were taken into account.

**Figure 12:**
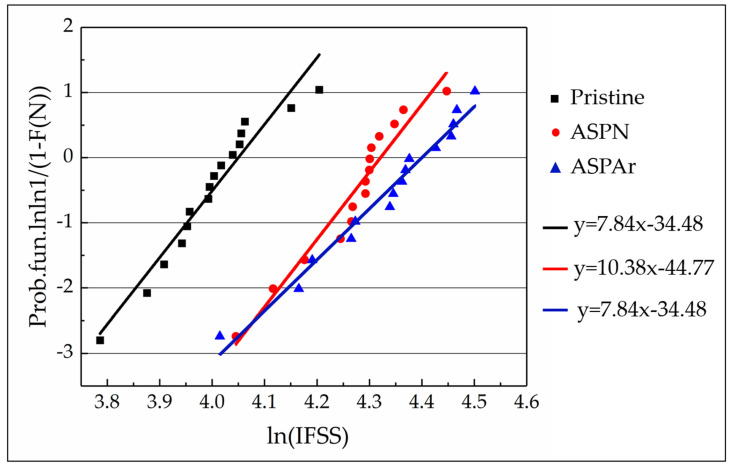
Weibull probability plots and linear fits for the push-out test data taken from the composites made with pristine, ASPN and ASPAr CFs.

For this reason, a Weibull distribution was adopted to statistically analyse the push-out test data. The Weibull plots and the fitted straight lines for the IFSS of composites made with three types of CFs are presented in Figure 12, and the results of the Weibull statistical analysis are summarised in Table 4. It can be seen that the Weibull plots were approximately linear, and all the fitting coefficient values R^2^ were over 93%, signifying that the IFSS of all composites followed a Weibull distribution. The Weibull scaling parameters (τ) of IFSS after ASP treatments followed the same trend with the average IFSS values. In addition, the Weibull plots of IFSS for composites with ASP-treated CFs were clearly separated from the pristine system, highlighting that the improvements in the IFSS values were statistically significant. Another observation related to Table 4 is that the ASPAr treatment slightly reduced the Weibull modulus, indicating that the measured data from ASPAr treatments possessed a slightly larger scattering.

It is widely accepted that the plasma surface modification of CFs is a complex physical and chemical process, which affects the interfacial adhesion through four mechanisms: (1) increasing the van der Waals binding by removing surface contaminations to provide a more intimate contact; (2) increasing mechanical interlocking sites by etching effect to roughen the surface and increase the surface area; (3) improving surface wettability of CFs to matrix by increasing the surface energy and polarity; and (4) enhancing chemical bonding though introducing functional groups on the fibre surface [32]. However, as the ASP-treated CFs exhibited a very similar surface morphology to the untreated fibres, as shown in Figure 5, the improved IFSS in this study was mainly derived from the nitrogen-containing functional groups on the CFs introduced by the ASP treatments and the oxygen-containing functional groups formed by the residual free radicals when re-exposed to air, which were shown to improve the surface hydrophilicity and reactivity of the CFs and hence improve the adhesion of CFs to the epoxy resin in composites. In addition to this, the mild etching effect from the advanced active screen plasma contributed to increasing the van der Waals binding and expanding the surface area without surface damage or strength degradation, as reported by our previous paper [15], which help to further improve the IFSS in resulting composites.

## 4. Summary and Conclusions

The study presented here explored the response of CFs to active screen plasma treatments with gas mixtures of N_2_-H_2_ (ASPN) and N_2_-H_2_-Ar (ASPAr) in terms of surface morphology, surface chemistry, water sorption behaviour and adhesion to epoxy resin, which are essential for the understanding of the interfacial adhesion mechanisms involved.

After the ASP treatments, the surfaces of CFs were enriched with nitrogen-containing and oxygen-containing functional groups, which increased the surface hydrophilicity and reactivity to epoxy resin. The introduction of argon in the nitrogen-hydrogen active screen plasma (i.e., ASPAr treatment) led to the formation of pyridinic nitrogen groups on CF surfaces and further increased the surface hydrophilicity because argon offered the potential of creating more active sites. However, the surface exhibited a hydrophobic recovery upon re-exposure to air for 60 days.

The IFSS between the ASP-treated CFs and epoxy resin improved by 32% when using ASPN-treated CFs and 41% when using ASPAr-treated CFs, as disclosed by single fibre push-out tests. Through the study of surface morphology, surface chemistry, water-sorption behaviour and the post-observation of pushed-out fibres, the interfacial adhesion enhancement mechanisms of ASP-treated CFs-reinforced epoxy composites could be mainly attributed to (1) the increased chemical bonding between the introduced functional groups on the fibre surface and the matrix; (2) the improved surface hydrophilicity of CFs; and (3) the enhanced van der Waals bonding due to the removal of surface contaminations.

## Figures and Tables

**Table 1:** Documented properties of HTA40 CFs [27].

Diameter (μm)	Strength (GPa)	Modulus (GPa)	Strain (%)	Density (g/cm^3^)	Sizing Type	Sizing Volume (%)
7	4.7	240	1.7	1.77	Epoxy	1.3

**Table 2:** XPS chemical quantification of pristine and ASP-treated CFs.

Sample Code	C 1s (%)	N 1s (%)	O 1s (%)
Pristine	81.1	1.4	17.5
ASPN	81.4	3.7	14.9
ASPAr	82.4	0.9	16.7

**Table 3:** Summary of DVS test results for pristine, ASPN and ASPAr CFs at 25 °C.

Sample Code	Dry Weight (mg)	Average Water Content (%)		Chemisorption (%)	Physisorption (%)
		Adsorption	Desorption		
Pristine	9.98	3.06	2.79	2.79	0.27
ASPN	9.75	3.74	3.57	3.57	0.17
ASPAr	10.02	4.46	4.28	4.28	0.17
ASPN (+60 days)	9.87	3.07	2.93	2.93	0.14
ASPAr (+60 days)	10.02	3.37	3.23	3.14	0.14

**Table 4:** Average interfacial shear strength and Weibull parameters for pristine, ASPN and ASPAr CFs.

Sample Code	*d* (μm)	Average IFSS (MPa)	Weibull Modulus (*m*)	Weibull Scaling Parameter, τ (MPa)	R^2^
Pristine	6.76 ± 0.32	55 ± 12	10.22	57	93.8
ASPN	6.62 ± 0.25	72 ± 9	10.36	75	93.1
ASPAr	6.63 ± 0.28	77 ± 10	9.80	81	97.3

## Data Availability

The data presented in this study are available upon request from the corresponding author.
